# Retraction: A Bmi1-miRNAs cross-talk modulates chemotherapy response to 5-fluorouracil in breast cancer cells

**DOI:** 10.1371/journal.pone.0319490

**Published:** 2025-02-24

**Authors:** The PLOS One Editors

After this article [1] was published, concerns were raised about Figures 1–3.

Specifically:

- The MDA-MB-231—BMI1 panels in Figures 1C and 3D appear similar when color levels are adjusted, despite representing different experimental conditions.
- The MDA-MB-231/sh panel in Figure 2A appears similar to the DADS+200b panel in Figure 4 of [2].
- Lanes 3–8 in the Actin panel in Figure 2B appear similar to lanes 2–7 in the Actin panel of Figure 2C, when rotated 180 degrees and with altered aspect ratio.
- In the Cyto-C panel of Figure 2C:
  - Lanes 1 and 6 appear similar.
  - There appears to be a vertical discontinuity between lanes 2 and 3.
- The areas surrounding multiple bands in the Caspase 9 and Caspase 7 panels of Figure 2C appear to be discontinuous with adjacent background areas when levels are adjusted.
- Lane 4 of the MCF-7/5-Fu - Actin panel and lane 1 of the MDA-MB-453 - Actin panel in Figure 3D appear similar, despite representing different experimental conditions.

The authors did not respond to queries about these concerns. In the absence of underlying data, the above issues remain unresolved.

In light of the above concerns, which call into question the integrity and reliability of these data, the *PLOS One* Editors retract this article.

All authors either did not respond directly or could not be reached.

Owing to the concerns about similarities with previously published content [2], published in 2013 by Elsevier, which is not offered under a CC BY license, the MDA-MB-231/sh panel of Figure 2A is therefore excluded from this article’s [1] license. At the time of retraction, the article [1] was republished to note this exclusion in the Figure 2 legend and the article’s copyright statement. Please refer to [2] for the correct copyright for this panel.
